# Unilateral Numbers Need Context: Reference Values for Single-Leg CMJ Performance Relative to Bilateral CMJ Capacity

**DOI:** 10.3390/sports14080323

**Published:** 2026-08-01

**Authors:** Karol Kruczek, Tim Gabbett, Jason Lake, Michał Nowak

**Affiliations:** 1 Sports Science Department, Next Generation Performance, 30-106 Kraków, Poland; 2 Gabbett Performance Solutions, Brisbane, QLD 4011, Australia; tim@gabbettperformance.com.au; 3 School of Sport, Science and Engineering, University of Chichester, Chichester PO19 6PE, UK; j.lake@chi.ac.uk; 4 Strength and Power Research Group, School of Medical and Health Sciences, Edith Cowan University, Joondalup, WA 6027, Australia; 5 Department of Physical Culture Sciences, Collegium Medicum Named Dr. Władysław Biegański, Jan Długosz University in Częstochowa, 42-200 Częstochowa, Poland; michal.nowak@ujd.edu.pl

**Keywords:** vertical jump, neuromuscular profiling, force plates, athlete monitoring, force–time analysis

## Abstract

The single-leg countermovement jump (SL CMJ) is widely used to assess limb-specific neuromuscular function, with interpretation commonly centred on inter-limb asymmetry. However, contextualising unilateral performance relative to bilateral capacity remains less clearly defined. This retrospective cross-sectional study aimed to develop exploratory descriptive benchmarks for SL CMJ kinetic, kinematic, and temporal variables expressed as a percentage of bilateral CMJ performance in a heterogeneous athletic cohort. A total of 348 athletes completed bilateral and unilateral CMJs using dual force plate sampling at 1000 Hz. The best trials were selected using the modified reactive strength index (mRSI), and averaged unilateral variables were expressed relative to bilateral potential. Unilateral-to-bilateral transfer was non-uniform across metric domains. Median relative jump height and the mRSI were 44.8% and 39.4%, respectively, whereas median average relative braking and propulsive force values were 76.0% and 75.4%, respectively. Gross impulse measures were retained to a greater extent than power- and rate-dependent variables, while temporal metrics showed prolonged unilateral phase durations. Exploratory sex-based analyses indicated trivial-to-small differences in selected variables; however, these findings should be interpreted cautiously because of unequal group sizes and the descriptive nature of the dataset. These results provide broad exploratory percentile benchmarks that may help practitioners contextualise SL CMJ performance relative to bilateral execution. However, the values should not be interpreted as universal clinical thresholds, injury-risk markers, or sport-specific normative standards without consideration of sex, sport discipline, competitive level, injury history, and longitudinal athlete context.

## 1. Introduction

The assessment of neuromuscular function via vertical jump testing has become a standard component of physiological profiling across high-performance sport, clinical sports medicine, military occupational fitness, and general population health monitoring. Specifically, the bilateral countermovement jump (CMJ) is widely regarded as the gold standard for evaluating lower-limb ballistic performance, neuromuscular fatigue/readiness, and adaptation to training loads [1,2,3,4,5,6]. This prevalence is likely driven by the favourable monitoring characteristics of the CMJ: it is quick to administer and minimally disruptive to training and can be repeated frequently [2]. The widespread adoption of force plate technology has further established the CMJ by allowing practitioners to move beyond simple outcome measures, such as jump height, to a granular analysis of kinetic and kinematic strategies across subphases [7]. While the bilateral CMJ effectively quantifies systemic force and power production, its ecological validity regarding sport-specific locomotion remains limited. Consequently, the single-leg countermovement jump (SL CMJ) has become an important diagnostic tool for functional assessment, as many decisive sporting actions, including acceleration, sprinting, changes in direction, and sport-specific manoeuvres, are governed by alternating single-limb support phases, while the metrics and indices derived from this test appear to reflect direction-specific neuromuscular qualities that are not fully captured by bilateral tasks and are therefore associated with these locomotor demands [8,9,10,11,12,13,14,15].

Unilateral testing is predicated on its capacity to isolate limb-specific performance, thereby exposing neuromuscular deficits that are frequently masked during bilateral execution through compensatory loading of the dominant or unaffected contralateral limb [16,17]. The SL CMJ is technically more demanding than its bilateral counterpart, requiring not only rapid force production but also enhanced proprioception, pelvic stability, and rotary control during the quiet, braking, propulsive, and landing phases [18]; ankle dorsiflexion mobility and postural stability have also been associated with CMJ performance and landing mechanics [19]. Given these added coordinative demands, unilateral protocols require stricter standardisation to reduce balance-related variability that can contaminate force–time metrics derived from the weighing phase [7]. Importantly, the selection of vertical jump assessments over horizontal jump alternatives is supported by recent biomechanical analyses [20,21]. Kotsifaki et al. [20] demonstrated that horizontal jumps are different in terms of work distribution between joints—historically the staple of return-to-sport (RTS) batteries—enable significant compensatory strategies via hip-dominant work. In contrast, vertical jump tasks necessitate significantly greater knee joint work, suggesting that the SL CMJ may be a more sensitive tool in some contexts for detecting residual deficits in knee-specific loading, particularly following anterior cruciate ligament (ACL) reconstruction [20]. Consequently, the utility of the SL CMJ extends to rehabilitation and RTS decision-making [22,23]. Specifically, inter-limb asymmetry quantified via SL CMJ metrics may serve as a vital biomarker for neuromuscular deficits [16]. Addressing these discrepancies may be clinically relevant, as restoring symmetry in force production and reactive strength is possibly critical for reducing re-injury rates [24]. Nevertheless, asymmetry magnitude and direction can be highly task-dependent and should be interpreted alongside absolute performance outputs and the underlying force–time strategy rather than as a standalone decision criterion [25]. In both sport and clinical practice, the SL CMJ is typically embedded within a broader athletic assessment battery and should be interpreted as a single input, rather than a standalone basis for, decision-making [26,27,28].

Although unilateral jump testing is well established in sports science and medicine, its practical interpretation should not rely on a single output in isolation. The existing biomechanical literature provides important insight into the mechanical and neuromuscular determinants of unilateral jump and landing performance; however, less attention has been directed toward how SL CMJ values can be contextualised within a broader assessment profile of the athlete or patient [7,29,30,31]. While increasingly accurate norms for various disciplines are available and specialists now analyse not only output measures and power-related variables but also detailed kinetic and kinematic strategies [32,33], these data are mainly reported as absolute values obtained from bilateral testing. Consequently, practitioners still do not know precisely how single-leg performance should relate to an athlete’s maximum neuromuscular capacity assessed bilaterally. Moreover, absolute SL CMJ values are difficult to categorise as optimal or deficient without reference to the athlete’s systemic efficiency. The bilateral CMJ inherently enables greater expression of maximal intent due to the increased base of support (two points of contact with force plates), which reduces the substantial balance constraints and quiet phase instability often observed in unilateral protocols [34]. From a measurement perspective, greater postural sway during single-leg quiet stance may compromise system weight estimation and event detection, thereby affecting other mechanical variables derived from the force–time signal [7].

The biomechanical distinctiveness of unilateral versus bilateral jumping has been well documented, with seminal work elucidating the kinetic and kinematic adjustments required during single-leg tasks, such as altered muscle activation patterns and joint-work distribution [35,36,37]. Aligning with these biomechanical challenges, Cohen et al. [38] highlighted the compounded mechanical demands of unilateral tasks, estimating that the active-leg load in a single-leg movement is approximately 1.62 times that of a bilateral execution. While the theoretical construct of the bilateral deficit (BD) has been widely explored to explain the reduced force output in bilateral tasks [39], this construct has primarily served mechanistic rather than diagnostic purposes. Biomechanical simulations have demonstrated that the reduced mechanical work per limb observed during bilateral jumps is primarily governed by the force–velocity relationship rather than solely by a suppression of neural drive, as the higher muscular shortening velocities achieved in two-leg executions inherently restrict maximal force production; nonetheless, a reduction in neural drive during simultaneous bilateral exertions has also been reported and may contribute to the deficit [40]. Furthermore, the initial distribution of system mass across two limbs causes the musculature to operate at a lower activation state during the early shortening phase, which limits total work output and underscores the unique, non-transferable mechanical constraints of unilateral tasks [41]. Conversely, the neuromuscular spectrum also encompasses bilateral facilitation (BF), which occurs when the biomechanical output during a bilateral CMJ exceeds the algebraic sum of independent unilateral trials [42]. Recent work relating unilateral and bilateral CMJ performance to force–velocity–power outcomes suggests that simple CMJ/SL CMJ metrics cannot be generalised to broader mechanical profiling or used to derive a practitioner-ready scaling factor [43]. Furthermore, evidence suggests limited consistency in the direction of inter-limb asymmetry across tasks [29], implying that bilateral performance serves as an imperfect proxy for unilateral capacity. Most existing SL CMJ research has been descriptive, focusing on task mechanics, inter-limb asymmetries, or selected performance outcomes. However, to our knowledge, no study has contextualised the full spectrum of SL CMJ-derived metrics relative to corresponding bilateral performance. This limits the ability to distinguish limb-specific underperformance from generally reduced systemic capacity and highlights the need for a more integrated interpretive framework.

Thus, the primary aim of this study was to develop descriptive percentile benchmarks for SL CMJ performance expressed relative to bilateral CMJ capacity in a heterogeneous athletic cohort under peak-state conditions, operationalised as the session with the highest session mean bilateral modified reactive strength index (mRSI). Rather than establishing universal normative thresholds, the present analysis sought to provide an exploratory reference framework for applied use that may help practitioners interpret whether SL CMJ outcomes are broadly aligned with an athlete’s bilateral performance profile. Unlike approaches focused solely on inter-limb asymmetry, this study examined the relationship between unilateral and bilateral execution across outcome, force, power, impulse, velocity, displacement, and temporal domains. Given the known biomechanical and neuromuscular differences in jump strategies between sexes, exploratory sex-based comparisons were also performed [44]. However, because of the unequal male-to-female sample distribution, these analyses were interpreted cautiously and were not intended to establish definitive sex-specific thresholds. Finally, this study aimed to improve the practical interpretability of unilateral jump testing by proposing a workflow for using unilateral-to-bilateral benchmarks alongside sport-specific context, clinical history, and longitudinal athlete monitoring.

## 2. Materials and Methods

### 2.1. Participants

The study used a retrospective cross-sectional design involving a heterogeneous cohort of athletes tested between August 2020 and February 2026. The final analytical sample included 348 athletes, comprising 274 men (age: 23.8 ± 6.8 years, height: 181.7 ± 9.8 cm, and body mass: 81.50 ± 16.00 kg) and 74 women (age: 22.9 ± 6.4 years, height: 167.1 ± 8.4 cm, and body mass: 65.98 ± 10.89 kg). Participants were active amateur, semi-professional, and professional athletes engaged in regular training and personalised, structured strength and conditioning training at the time of testing. All assessments were conducted at a single high-performance training centre under the supervision of certified strength and conditioning coaches. In this retrospective analysis of routinely collected force plate monitoring data, no a priori sample-size calculation was performed; instead, the analytical sample comprised all athletes whose records satisfied the predefined adherence and quality criteria during the monitoring period.

Because the dataset originated from routine applied performance monitoring rather than a prospective research project, several contextual variables were not systematically collected or controlled. Specifically, detailed information regarding sporting discipline, competitive level, season phase, previous musculoskeletal injuries, surgical history, limb dominance, and long-term functional restrictions was not consistently recorded across the full retrospective database. Consequently, these variables were unavailable for subgroup analyses and should be considered when interpreting the findings.

Similarly, no attempt was made to standardise or control several factors known to influence neuromuscular performance. Information regarding athlete wellness status, psychological readiness, recent training load, accumulated fatigue, footwear, nutritional practices, caffeine intake, supplementation strategies, hydration status, or—in female athletes—menstrual cycle phase was not systematically collected. As such, the dataset reflects the realities of routine field-based athlete monitoring rather than tightly controlled laboratory testing conditions.

The only criterion that routinely excluded athletes from testing was the presence of pain or musculoskeletal discomfort that could compromise safe maximal-effort jumping. Athletes reporting acute pain, injury-related symptoms, or movement-related discomfort at the time of testing were not assessed until symptoms had resolved sufficiently to permit maximal voluntary effort. This criterion was applied because the purpose of the testing process was to evaluate maximal neuromuscular performance under conditions that allowed for unrestricted movement execution.

### 2.2. Ethical Considerations

The study was conducted in accordance with the Declaration of Helsinki and received a positive opinion from the Research Ethics Committee at Jan Długosz University in Częstochowa (Resolution No. KE-U/9/2026, 19 January 2026). The dataset was derived from routinely performed assessments conducted before the present retrospective analysis was designed. At the time of routine testing, athletes were informed that de-identified performance data could be used for scientific and quality-improvement analyses and that they could refuse the secondary use of their data without sporting or organisational consequences. The Research Ethics Committee approval therefore applied specifically to the retrospective secondary analysis of masked data, rather than to a prospective intervention or additional data collection. All data were anonymised before analysis to protect participant identity.

### 2.3. Testing Procedures

A standardised testing protocol was implemented to ensure longitudinal consistency and data integrity. Testing was consistently scheduled as the first component of the training session, immediately following a standardised warm-up protocol. The warm-up consisted of three progressive blocks: (1) general aerobic activity (cycling or running) lasting ~5–8 min, (2) dynamic bodyweight exercises (e.g., glute bridges, lunges, and calf raises), and (3) plyometric actions (rebound jumps and three submaximal CMJs for familiarisation—if necessary), followed by a 90 s rest period before testing (Supplementary Table S1). The assessment protocol commenced with the CMJ, executed in a cluster-set configuration consisting of two series of three repetitions (3 + 3), separated by a 60–90 s recovery interval to sustain maximal output [45]. Following the bilateral assessment, the SL CMJ was performed, comprising two series of three repetitions per limb, with the same 60–90 s inter-set rest period used to facilitate sufficient phosphocreatine resynthesis to sustain maximal power expression [46]. To mitigate acute fatigue while maintaining neural potentiation within sets, a 5–10 s inter-repetition rest was incorporated into the protocol. The adoption of these temporal ranges, rather than fixed time points, accounts for the ecological constraints of applied monitoring, accommodating inevitable logistical variations such as equipment recalibration, athlete feedback, or immediate data verification. To isolate lower-limb neuromuscular performance and eliminate arm swing momentum, participants were required to maintain a hands-on-hips position throughout both protocols. Specific technical constraints were applied to the SL CMJ to minimise compensatory strategies: the non-stance limb was maintained in a neutral position with the knee flexed directly inferior to the pelvis, preventing any active swinging motion that could artificially enhance jump height. To maximise comfort and coordination, participants were permitted to self-select the starting limb for the initial unilateral trial. Furthermore, to ensure safety and standardise the deceleration phase, all unilateral jumps were landed bilaterally. Each trial was initiated following the manufacturer-defined audiovisual cue provided by the tablet interface, which indicated system readiness after a stable quiet standing phase had been registered. System weight calculation and movement-onset detection were based on the manufacturer’s default settings, and no additional filtering was applied to the raw force–time data. The instruction provided was: “jump as high and as fast as possible.” Feedback was given after each attempt in the form of jump height, and the force plates were zeroed regularly between participants to ensure calibration stability. Vertical ground reaction forces were sampled at 1000 Hz using dual wireless force plates (Hawkin Dynamics, Westbrook, ME, USA). The validity and reliability of this hardware in comparison with industry-standard wired systems have been previously established [47,48]. Throughout the six-year data collection period, the proprietary software and firmware were updated periodically in accordance with manufacturer releases. To ensure high consistency, all strength and conditioning coaches supervising the testing sessions underwent the same training procedure and adhered to a standardised protocol script regarding verbal encouragement and technical cues. In instances where the diagnostic protocol was expanded in real-time (e.g., additional trials due to technical errors or coaching decisions), the dataset was subsequently reduced during the data filtering phase to adhere to the strict inclusion criteria described below.

### 2.4. Data Extraction and Filtering Logic

**Figure 1:**
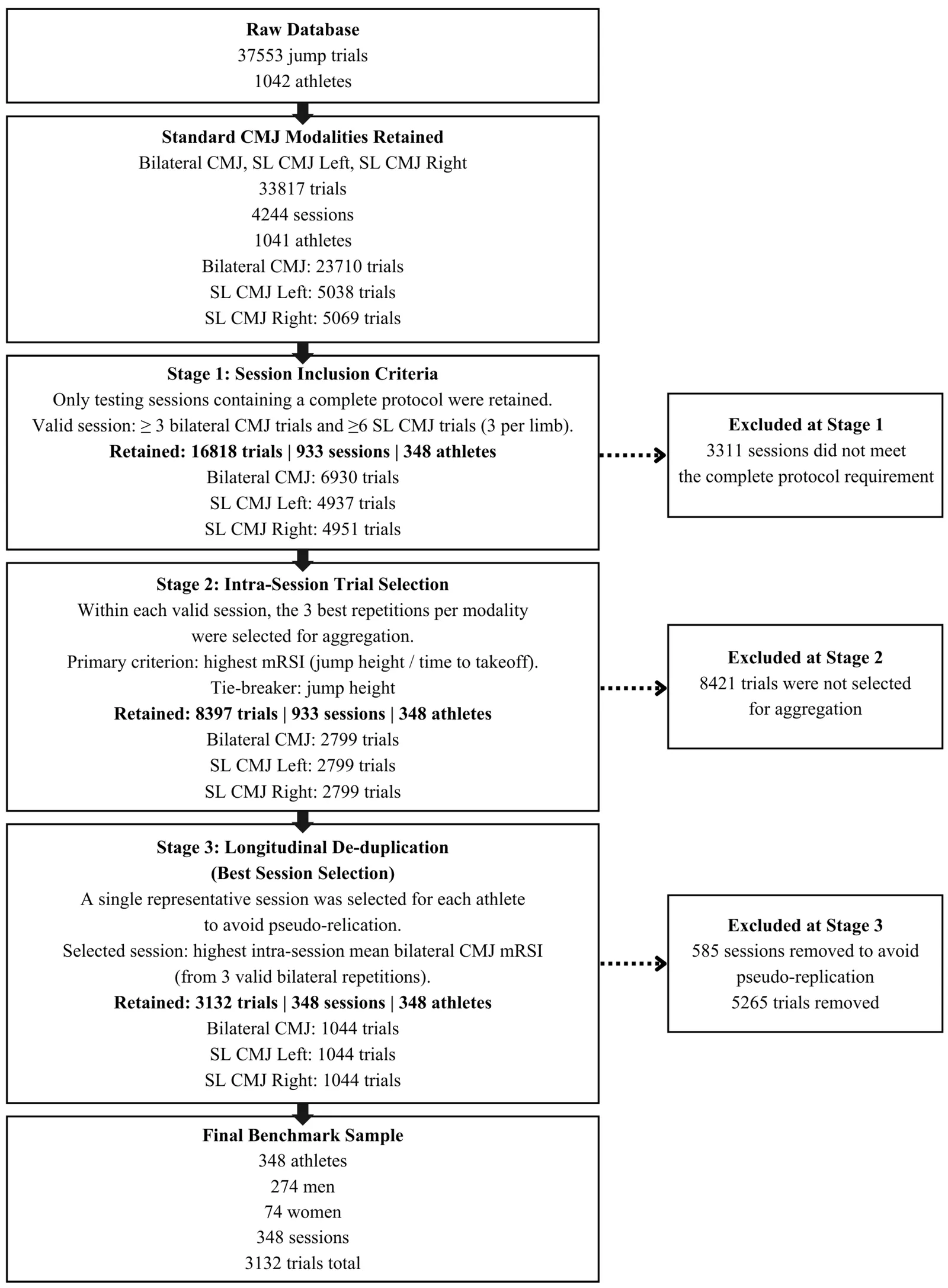
Flow chart of athlete, session, and trial selection. Legend: CMJ—countermovement jump; SL—single-leg; mRSI—modified reactive strength index.

Force–time data were extracted via the manufacturer’s application programming interface and subsequently processed using a custom Python 3.14.6 script developed by the research team. To ensure transparency and reproducibility, the complete raw dataset and the processing code have been deposited in a public repository. To ensure the integrity of the reference dataset and eliminate non-representative trials, a rigorous multi-stage filtering protocol (Figure 1) was applied to the raw dataset. Comprehensive operational definitions and calculation parameters for all selected force–time metrics are detailed in Supplementary Table S2.

To guarantee sufficient data density for reliable averaging, only testing sessions containing a complete protocol were retained. A valid session was defined as one including a minimum of three completed bilateral CMJ trials and a minimum of six SL CMJ trials (three repetitions per limb). Sessions failing to meet this volume threshold were excluded from the analysis. Within each valid session, the three best repetitions for each jump modality (“Bilateral CMJ”, “SL CMJ Left”, “SL CMJ Right”) were selected for aggregation. The primary criterion for trial selection was the highest mRSI, defined as jump height divided by time to takeoff. This metric was prioritised to isolate trials exhibiting the highest neuromuscular proficiency under temporal constraints, rather than maximal positive centre-of-mass displacement alone. This selection strategy mirrors the demands of dynamic, sport-specific tasks, where the window for force application is often restricted. Indeed, meta-analytic evidence indicates that the mRSI shares significant associations with critical time-dependent performance metrics—including linear sprint, acceleration, and change in direction effectiveness—thereby serving as a more ecologically valid proxy for sport-specific explosiveness than jump height [49]. In the event of identical mRSI values, jump height served as the secondary tie-breaking criterion. For athletes with longitudinal data spanning multiple testing dates, a single representative session was selected to avoid pseudo-replication. The session displaying the highest intra-session mean bilateral CMJ mRSI (derived from three valid repetitions) was chosen as the athlete’s peak potential baseline for descriptive reference classification. These reference values represent idealised physiological states and may overestimate daily average readiness expectations.

### 2.5. Calculation of Bilateral Potential

Following the filtering process (N = 348), kinetic and kinematic metrics were averaged for bilateral (*Mean_Bilateral_*), unilateral (*Mean_Unilateral_*), left (*Mean_Left_*), and right (*Mean_Right_*) trials. To establish a unified metric for unilateral capacity, a composite unilateral score was calculated as the arithmetic mean of the limb-specific values:

*Mean_Unilateral_* = (*Mean_Left_* + *Mean_Right_*)/2 (1)

Subsequently, each unilateral metric was expressed as a percentage of the athlete’s bilateral potential using the following equation:

%*Bilateral* = (*Mean_Unilateral_*/*Mean_Bilateral_*)⋅100 (2)

### 2.6. Statistical Analysis

Descriptive statistics were calculated for all derived variables and included the mean, standard deviation (SD), and t-based 95% confidence intervals (95% CI). Consistent with recommended statistical reporting practice, the mean and SD were reported as separate summary parameters rather than in mean ± SD format [50]. Normality of each athlete-level unilateral-to-bilateral percentage variable was assessed using the Shapiro–Wilk test at α = 0.05. Tests were performed for the pooled cohort and are reported in Supplementary Table S6. To establish practitioner-oriented reference values, percentile values were calculated at the 3rd, 10th, 25th, 50th, 75th, 90th, and 97th percentiles. For benchmark interpretation, the 50th percentile (median) was adopted as the primary measure of central tendency because the heterogeneous composition of the cohort increased the likelihood of skewed distributions and influential outliers. However, the mean and SD were additionally retained as part of a dual-reporting strategy serving two complementary purposes: first, comparison of the mean and median provides a practical indication of distributional asymmetry and potential outlier influence across force–time variables; second, reporting mean and dispersion parameters preserves compatibility with conventional aggregate-data meta-analytic workflows for continuous outcomes [50,51]. For temporal metrics (e.g., braking phase duration, time to takeoff), percentile ranking was reverse-scored so that shorter durations corresponded to higher reference ranks and all percentile outputs could be interpreted in the same direction, with higher percentile ranks reflecting superior performance. For metrics that were negative by sign convention (e.g., peak braking velocity, stiffness), absolute magnitudes were analysed to facilitate standardised interpretation. Relative variability was quantified using the coefficient of variation, expressed as a percentage (CV%), and calculated as SD/mean⋅100.

Between-sex differences were evaluated using two-tailed Welch’s independent-samples *t*-tests, selected a priori because group sizes were unequal and homogeneity of variance could not be assumed. Statistical significance was set at *p* < 0.05. No formal adjustment for multiple comparisons was applied because the sex-based analyses were secondary and exploratory relative to the primary benchmarks. Standardised mean differences were expressed as Cohen’s d with 95% confidence intervals and interpreted using the following thresholds: trivial (<0.2), small (0.2–0.49), medium (0.5–0.79), and large (≥0.8) [52]. To complement mean-based comparisons, supporting rank-based between-sex differences were additionally examined using Mann–Whitney U tests, with effect magnitude expressed as rank-biserial correlation (r) and its 95% confidence interval. Following the session- and trial-level filtering steps described above, analyses were conducted on complete cases only and no missing-data imputation was required. All statistical procedures were performed in Python using pandas, numpy, scipy, pingouin, matplotlib, requests, openpyxl, and statsmodels.

### 2.7. Data Availability

To ensure transparency and facilitate the replication of our findings, the complete de-identified dataset and the custom Python processing scripts used in this study have been deposited in the Zenodo repository and are freely accessible via the following persistent identifier: https://doi.org/10.5281/zenodo.18710843.

## 3. Results

### 3.1. Reference Distribution of Unilateral-to-Bilateral Transfer

Exploratory reference values for SL CMJ variables, expressed as a percentage of bilateral capacity, are summarised in Table 1 (N = 348). Across the dataset, a non-uniform pattern of unilateral-to-bilateral transfer was observed. Outcome measures displayed the largest reductions in the unilateral condition, rate-dependent variables and power metrics showed moderate reductions, whereas several force-based variables and gross impulse measures exhibited comparatively higher transfer values. Temporal variables indicated a systematic alteration in phase duration relative to the bilateral condition.

Importantly, dispersion differed by metric type. Force-based variables generally exhibited tighter typical ranges, while strategy variables (e.g., stiffness, braking phase duration, braking rate of force development) showed substantially greater between-athlete spread. Collectively, these results indicate that unilateral performance cannot be inferred using a simple half-of-bilateral heuristic and instead reflects distinct profiles across all subgroups of force–time-derived variables (Table 1).

Table 1 presents pooled reference values for the entire cohort, irrespective of sex, as all SL CMJ variables were reported exclusively as individual percentage-normalised values, with no absolute performance outputs presented.

The primary outcome measures showed lower unilateral transfer than most kinetic variables (Table 1). The typical athlete’s unilateral jump height was below half that of the bilateral outcome, and SL CMJ mRSI was lower still, indicating that time-constrained performance is disproportionately reduced in the unilateral condition. Percentile distributions highlighted substantial between-athlete dispersion for both outcomes. This variability was further reflected in the CVs, which reached 12.9% for jump height and 15.7% for the mRSI, indicating moderate to relatively high variability across the cohort. These findings support the use of percentile-based interpretation rather than reliance on single-point estimates.

### 3.2. Braking Phase Force and Power

Braking-phase metrics displayed a clear domain pattern: force retention surpassed power transfer, and slope-derived variables exhibited the largest reductions and the broadest dispersion (Table 1). CVs were 7.3–8.1% for braking force metrics (average and peak; absolute and relative), 15.3–16.6% for braking power metrics (average and peak; absolute and relative), and 28.5% for braking rate of force development. At the median level, the force–power gap in braking was approximately 17–23 percentage points. Unilateral execution maintained average and peak braking force to a greater extent than braking power and braking rate of force development.

### 3.3. Propulsive Phase Force and Power

Propulsive phase results mirrored the braking phase pattern. Force-derived metrics clustered at higher retention transfer values than power-derived metrics, again yielding a median force–power difference of 16–22 percentage points (Table 1). CVs were 5.2–8.0% for propulsive force metrics (average and peak; absolute and relative), 7.7–8.3% for propulsive power metrics (average and peak; absolute and relative), and 4.9–6.6% for velocity metrics (propulsive average and peak, takeoff velocity).

### 3.4. The Mechanical Influence of System Weight on Impulse

Impulse metrics revealed one of the most distinctive findings in the dataset: gross impulse measures (including system weight) were substantially higher than net impulse measures (impulse above system weight). At the median, gross–net separations were consistently ~28 percentage points across phases, including braking, propulsion, and the cumulative positive impulse expressions (Table 1). Relative variability was modest for positive impulse (gross: CV = 7.1%; net: CV = 7.3%) and the lowest for propulsive impulse metrics (gross: CV = 6.6%; net: CV = 6.8%) but was notably higher for braking impulse metrics, particularly in their net expressions (gross: CV = 9.6%; net: CV = 12.8%).

### 3.5. Kinetic and Kinematic Description of Jump Strategy

Temporal metrics indicated systematic adjustments in unilateral execution (Table 1). Braking and propulsive phase durations were generally prolonged relative to the bilateral condition, contributing to a longer overall time to takeoff. Movement strategy variables further indicated heterogeneity in unilateral execution. During the SL CMJ, countermovement depth was shallower than the bilateral equivalent, whereas vertical stiffness showed high dispersion. Average and peak velocities for both braking and propulsion settled within a median range of 67–70% of bilateral potential, and both absolute and relative force at minimal displacement aligned with this tier (Table 1). CVs were 10.0–15.1% for temporal variables (unweighting, braking, propulsive duration, and time to takeoff), 14.5% for countermovement depth and 20.6% for stiffness, 12.1–12.7% for braking velocity metrics, 4.9–6.6% for propulsive/peak/takeoff velocity metrics, and 8.0–8.2% for force at minimal displacement (absolute and relative).

### 3.6. Sex-Based Comparative Analysis

Exploratory sex-based comparisons are presented in Supplementary Table S3, and sex-stratified descriptive percentiles for all variables are provided in Supplementary Table S4 (men) and Table S5 (women). Several statistically significant differences were observed; however, effect sizes were trivial-to-small, and the overall domain-specific pattern of unilateral-to-bilateral transfer was consistent across male and female athletes. In general, men exhibited slightly higher transfer in outcome measures (jump height and mRSI), whereas women showed slightly higher transfer in selected force metrics, particularly braking-related variables. By contrast, propulsive net impulse expression and impulse ratio tended to favour males. Given the unequal group sizes and the heterogeneous sport composition of the cohort, these comparisons should be interpreted as descriptive and hypothesis-generating rather than as sex-specific normative thresholds.

## 4. Discussion

The present study contributes to the literature by addressing the limited availability of reference-based interpretive frameworks for SL CMJ assessment that may facilitate more efficient and informative athlete profiling. Although the SL CMJ is well established in sports science and applied practice, its performance metrics remain less clearly contextualised relative to bilateral capacity. Against this background, the primary aim of this investigation was to establish reference data for SL CMJ variables expressed as a percentage of bilateral potential, thereby providing practitioners with a comprehensive framework for interpreting unilateral vertical jump execution. The most significant finding of this study is the clear dissociation between output and strategy variables. While the theoretical construct of the BD suggests that the sum of unilateral forces should exceed bilateral force production [53], our data indicate that this force surplus does not translate linearly into bilateral outcomes such as jump height or the mRSI. The median relative jump height of 44.8% demonstrates that the typical athlete cannot replicate half of their bilateral displacement on a single limb. Consequently, because the algebraic sum of unilateral performances falls short of the bilateral maximum, our data suggest that outcome metrics in the CMJ are heavily governed by BF [42]. This finding also challenges the simplistic assumption often held in coaching practice that a balanced athlete should achieve 50% of their bilateral score. Instead, it suggests that unilateral ballistic performance is constrained not by force generation capacity but by the velocity-specific demands of projecting the entire body mass with a single kinetic chain.

### 4.1. The Mechanical Consequence of Unilateral Loading

To interpret unilateral deficits correctly, one must first account for the role of system weight (the product of body mass and gravitational acceleration, estimated during the initial quiet-standing phase of the force-plate assessment). Unlike the bilateral condition, where the gravitational load is distributed between two limbs, the stance-leg in the SL CMJ must counteract almost the entirety of the body mass solely to initiate movement. Consequently, the obligatory mechanical demand is effectively nearly doubled before any unweighting, braking or propulsion occurs. This gravitational constraint creates a natural inflation of gross force, impulse and power metrics when expressed as a percentage of bilateral potential. Because these metrics incorporate system weight, they do not start from a zero baseline but from the magnitude of the athlete’s body mass. Therefore, a relative force value significantly exceeding half of the bilateral output is not necessarily indicative of a physiological BD but rather a mechanical necessity: the limb must generate sufficient force to overcome the entire system weight, or the athlete cannot initiate upward acceleration and leave the ground. This phenomenon is most clearly illustrated by the divergence between gross impulse and net impulse variables. Gross impulse values, which include the impulse required to support body mass, therefore remained high, whereas net impulse values, which represent the impulse contributing to changes in momentum, were substantially lower and more closely aligned with jump height ratios. Thus, the SL CMJ should be primarily viewed as a test of the limb’s ability to tolerate nearly a twofold load, rather than a simple fraction of bilateral performance.

### 4.2. Compensatory Mechanisms: Mitigating Power and Explosiveness Deficits via Temporal Extension

Accordingly, the findings of the current study should be interpreted in light of the basic, obligatory mechanical constraint imposed by unilateral loading, under which impulse maximisation appears to depend on specific temporal strategies adopted by the neuromuscular system. The dissociation between high force retention and reduced power output provides a novel insight into the compensatory strategies utilised during unilateral tasks. The present findings revealed that while average relative propulsive force was maintained at high levels (median: 75.4%), average relative propulsive power dropped considerably (median: 53.2%). This finding aligns with the fundamental force–velocity relationship, where an increase in external load—in this case, the relative mass supported by a single stance limb—necessitates a proportional decrease in propulsive velocity. Notably, similar constraints are evident in negative velocity. The observed reduction in descent momentum likely stems from the inherent difficulty of rapid unweighting over a reduced base of support. In this unstable environment, balance requirements likely supersede the intent for maximum negative acceleration, preventing the athlete from aggressively entering the braking phase. Consequently, this safety-first strategy may compromise the magnitude of eccentric loading, thereby diminishing the potentiation derived from the stretch-shortening cycle (SSC) [54]. To overcome this power deficit, athletes instinctively adopted a strategy of temporal elongation. The median time to takeoff reached 113.5% alongside a propulsive net impulse of 67.1% (because impulse represents the net force–time integral, increasing this value through duration rather than force magnitude may indicate a suboptimal strategy for time-limited actions or an undesirable training adaptation), indicating that to generate sufficient momentum, athletes are compelled to apply force for a significantly longer duration. This highlights a mechanical divergence between tasks; whereas the bilateral CMJ facilitates power expression, the SL CMJ may provide a demanding assessment of the capacity to apply force effectively under heightened coordination and mechanical demands [34].

### 4.3. Implications for Rehabilitation

Recent meta-analyses and systematic reviews support the clinical utility of vertical jump testing, including unilateral variants, indicating that force plate-derived metrics provide valuable and responsive data for discriminating functional deficits and tracking recovery during rehabilitation [5,6]. Whereas performance monitoring often prioritises individual jump outputs to index fatigue or readiness [4], sports medicine requires a deeper, mechanism-informed interpretation rooted in injury aetiology; accordingly, braking-phase metrics may provide important context for RTS decision-making, as many severe non-contact lower-limb injuries occur during single-leg braking, landing, or rapid transition manoeuvres, including ACL injuries in football and Achilles tendon ruptures in basketball [55,56]. The magnitudes of the average relative braking force (median: 76.0%) and relative force at minimal displacement (median: 69.8%) are a direct consequence of the unilateral limb accommodating almost the entire system mass plus inertial forces. These values indicate that during the negative and amortisation phases of the SL CMJ, the knee extensors are subjected to a disproportionately high relative load compared with bilateral conditions. Practically, this implies that an athlete who appears symmetrical in low-velocity strength tests (e.g., concentric-oriented isokinetic dynamometry) may still perform poorly during the SL CMJ because they lack the specific eccentric and quasi-isometric capacity required to effectively dampen centre of mass momentum (body mass⋅negative velocity). Supporting this, O’Malley et al. [22] found that isolated strength tests were insufficient, whereas combining them with the SL CMJ distinguished ACL-reconstructed patients from healthy controls with 89% accuracy. While the bilateral CMJ may mask deceleration deficits via compensatory load distribution, the unilateral variant demonstrates that effective momentum attenuation is compromised if the athlete cannot tolerate the obligatory mechanical demands of moving their body mass. Consequently, the SL CMJ should be viewed not merely as a performance test but as a high-demand eccentric stress for the entire lower-limb kinetic chain, specifically accentuating the knee extensor mechanism because of the greater knee-joint work demands compared with horizontal tasks [20]. However, the clinical utility of the presented benchmark data extends beyond the braking capacity. The analysis provides critical insight into propulsive kinetic outputs and the kinematic description of movement strategy. Specifically, metrics such as countermovement depth and peak braking velocity may serve as valuable indicators of the athlete’s neuromuscular state [23]. In a clinical setting, deviations in these parameters may reflect underlying kinesiophobia, restrictions in functional range of motion, or deficits in intermuscular coordination required for effective unweighting. Therefore, a comprehensive interpretation of SL CMJ performance must integrate a wide spectrum of mechanical variables to fully capture the athlete’s potential readiness for the dynamic demands of sport.

### 4.4. Inter-Individual Variability and Metric Dispersion Profile

A methodological examination of metric dispersion reveals a clear distinction between the stability of performance outcomes and the variability of execution strategies. This divergence is observable even within the primary outcome measures; jump height remained relatively stable (SD = 5.66 percentage points (pp); CV = 12.9%), whereas the mRSI—an index inherently constrained by time to takeoff—exhibited slightly greater variability (SD = 6.16 pp; CV = 15.7%). Variability became more pronounced when isolating specific strategy variables, particularly vertical stiffness (SD = 20.30 pp; CV = 20.6%) and braking rate of force development (SD = 12.09 pp; CV = 28.5%), whereas braking phase duration showed high absolute but only moderate relative variability (SD = 18.06 pp; CV = 14.1%). This pattern likely stems from two factors. First, rate-dependent variables are mathematically more sensitive to signal noise and derivative-based calculations than aggregate measures such as impulse [57,58]. Second, the data are consistent with the principle of motor equivalence within a redundant neuromuscular system; the convergence on a relatively narrow range of jump heights despite broader variance in force–time and spatiotemporal characteristics suggests that athletes may achieve similar functional outcomes through different mechanical strategies, including varying degrees of system compliance [59].

### 4.5. Sex-Specific Neuromuscular Strategies

The comparative analysis between sexes highlights nuances in how men and women manage these unilateral constraints, though these differences should be interpreted with caution. In the present retrospective database, female athletes represented only approximately 21% of the analysed population; therefore, sex-specific observations should be considered hypothesis-generating rather than definitive empirical evidence. While male athletes demonstrated a slightly superior transfer efficiency for jump height and the mRSI, and female athletes exhibited higher force metrics, the magnitude of these differences was generally trivial-to-small. This suggests that while sex-specific strategies may exist—with females potentially relying slightly more on force-dominant mechanisms to compensate for reactive strength deficits—the fundamental biomechanical determinants of the SL CMJ appear to remain consistent across both populations. Therefore, while practitioners should be aware of these tendencies during profiling, the reference data suggest that radical divergence in training interventions or testing protocols based solely on sex is likely unwarranted. Both groups face the same primary constraint: the need to express high force rapidly to overcome the effectively increased relative load resulting from the reduction to a single point of support.

### 4.6. Practical Applications

The evaluative framework presented in this study, which scales SL CMJ performance relative to bilateral performance, may provide an additional interpretative layer for vertical jump testing in both high-performance and clinical environments by offering further context for unilateral assessment. Practitioners should recognise that the transfer of bilateral proficiency is non-uniform across the kinetic and kinematic spectrum. A key interpretive consideration is the recognition of the obligatory mechanical load imposed by the athlete’s body mass. Unlike bilateral tasks where load is distributed, the unilateral limb must counteract the entirety of the system weight, almost doubling the relative load per limb. Consequently, practitioners should anticipate naturally high unilateral-to-bilateral ratios (often exceeding 70–75%) in gross force and impulse variables. This elevation reflects a mechanical necessity rather than a BD and should not be misidentified as superior unilateral capability. The authors advocate for a redefinition of functional vertical jump assessment, encouraging professionals to refine their diagnostic protocols or adopt the standardised methodology detailed in the present study to ensure data integrity. By benchmarking individual results against these reference data derived from a six-year retrospective dataset, coaches and clinicians can more precisely orient assessment feedback, training interventions and rehabilitation pathways. Although the present dataset is heterogeneous, this methodological approach may be applied within more homogeneous sport-, sex-, or injury-specific cohorts to develop context-specific normative profiles and better identify jump strategies related to typical adaptations or injury-related constraints.

### 4.7. Limitations and Future Research Directions

The findings of this investigation should be interpreted with consideration of the breadth and pragmatic origin of the reference dataset. First, the inclusion of athletes across multiple backgrounds supports the relevance of the benchmark values for a broad athletic population but reduces the resolution with which subgroup-specific biomechanical signatures can be identified. Accordingly, the pooled estimates may attenuate distinct neuromuscular strategies present within particular athletic subpopulations and contribute to residual variability. In addition, the primary reference table presented in this article integrates data from both male and female athletes. Given the unequal sex distribution within the final sample (74 women and 274 men), the pooled values are likely more strongly influenced by male performance patterns and should not be interpreted as equally weighted sex-neutral reference standards. To address this limitation and support more context-specific interpretation, separate sex-stratified reference tables are provided in the Supplementary Materials.

Second, because the database was assembled from existing assessments performed in a real-world performance environment rather than from a protocol designed a priori to isolate causal determinants of SL CMJ performance, the reported benchmarks should primarily be interpreted as field-derived reference values. They should not be used for causal inference but rather as practical reference points to inform coaching and clinical decision-making. Furthermore, the standardised testing protocol administered the SL CMJ after the bilateral assessment. Although rest intervals were provided, the potential for residual intra-session fatigue or attentional decrement cannot be fully excluded. To mitigate this order effect and ensure optimal neural drive for both modalities, future normative studies should consider separating unilateral and bilateral testing sessions by 24–48 h, although this may not always be practical in athletic populations.

Third, a critical interpretive caveat concerns the data curation strategy. By strictly isolating the single session in which each athlete achieved their highest observed bilateral mRSI (average score from the three best repetitions), the dataset is intentionally biased toward evaluating maximal neuromuscular capacity. Additionally, this selection criterion—predicated on identifying the peak bilateral performance—may introduce a specific bias: an athlete’s peak bilateral readiness does not necessarily coincide temporally with their peak unilateral capability. Consequently, calculating ratios based on the best bilateral day may juxtapose a maximal bilateral value against a submaximal unilateral output, thereby artificially inflating the BD and resulting in reference ratios that are slightly underestimated relative to the athlete’s theoretical potential. While this anchors the benchmark to peak bilateral performance, the resulting ratios may not represent those expected during routine training phases characterised by accumulated fatigue.

Building on these benchmark baselines, future research should focus on several primary pathways. First, longitudinal investigations are required to determine whether the ratio of unilateral-to-bilateral performance represents a stable neuromuscular trait or a fluctuating state sensitive to specific forms of fatigue, including acute and chronic fatigue responses. Second, prospective studies are warranted to examine whether falling below specific thresholds of bilateral potential reflects a mechanically inefficient neuromuscular profile. Such relative deficits may hypothetically predispose athletes to a higher incidence of both cumulative overuse and acute non-contact injuries during high-demand sport-specific manoeuvres. Third, although the present study established benchmarks for limb performance in isolation, future work should integrate these reference values with inter-limb asymmetry indices to determine the potential cost of asymmetry on total bilateral output and on the unilateral-to-bilateral ratio itself. Finally, subsequent investigations should categorise these broad baseline values across more homogeneous cohorts to identify distinct neuromuscular adaptations dictated by sporting discipline, positional demands, clinical history, or predominant training modalities. Establishing such population-specific normative datasets may maximise the diagnostic precision of the unilateral-to-bilateral ratio, enabling practitioners to better tailor rehabilitative and performance interventions to the unique biomechanical characteristics of targeted athletic subpopulations.

## 5. Conclusions

This study addresses a gap in neuromuscular assessment by providing one of the first large-scale percentile-based reference datasets for multiple unilateral-to-bilateral ratio metrics derived from force plate CMJ assessment. By establishing that reference unilateral output is task-dependent—ranging from a median ~39% for the mRSI to a median ~94% for stiffness—the findings challenge the utility of the intuitive 50% rule of thumb in applied diagnostics. The presented data demonstrate that the relationship between unilateral and bilateral performance is not a fixed constant but rather operates along a broad biomechanical continuum, stretching from pronounced BF in outcome variables to a substantial BD governing movement strategy parameters. Consequently, this research provides practitioners with an exploratory, percentile-based descriptive framework for general athletic populations to contextualise SL CMJ performance, facilitating the distinction between obligatory mechanical constraints and neuromuscular deficits in rehabilitation and performance settings.

## Figures and Tables

**Table 1:** Descriptive statistics and percentile distribution of neuromuscular performance parameters in the SL CMJ expressed as a percentage of bilateral capacity.

Metric	N	Mean	95% CI Lower	95% CI Upper	SD	Minimum	P3	P10	P25	P50 Median	P75	P90	P97	Maximum
**Jump Height [m]**	348	44.3%	43.7%	44.9%	5.7%	22.7%	34.2%	36.8%	40.4%	44.8%	48.5%	51.0%	53.8%	58.2%
**mRSI [a.u.]**	348	39.4%	38.7%	40.0%	6.2%	20.9%	26.9%	31.6%	35.7%	39.4%	43.5%	46.4%	50.5%	56.3%
**Jump Momentum [kg**·**m/s]**	348	66.5%	66.0%	67.0%	4.5%	47.4%	58.5%	60.8%	63.5%	67.0%	69.6%	71.5%	73.5%	91.2%
**Unweighting Phase Duration [s]**	348	97.0%	95.5%	98.5%	14.6%	157.6%	127.5%	116.0%	104.5%	94.9%	86.4%	80.1%	76.2%	70.0%
**Braking Phase Duration [s]**	348	127.8%	125.9%	129.7%	18.1%	187.5%	164.4%	150.5%	138.9%	127.5%	115.1%	106.6%	97.2%	77.0%
**Propulsive Phase Duration [s]**	348	126.2%	124.8%	127.5%	12.8%	183.6%	147.9%	141.2%	133.8%	126.5%	118.5%	111.0%	102.1%	90.2%
**Time to Takeoff [s]**	348	113.9%	112.7%	115.1%	11.4%	159.5%	138.3%	127.8%	120.0%	113.5%	106.5%	100.8%	94.0%	87.0%
**Countermovement Depth [m]**	348	73.3%	72.2%	74.4%	10.6%	38.7%	53.2%	59.2%	66.6%	73.8%	79.4%	85.0%	96.3%	104.5%
**Stiffness [N/m]**	348	98.8%	96.6%	100.9%	20.3%	56.1%	69.1%	77.6%	85.8%	94.5%	107.2%	125.5%	142.9%	219.8%
**Force at Minimal Displacement [N]**	348	70.0%	69.4%	70.6%	5.8%	50.6%	59.3%	62.9%	66.2%	69.9%	73.2%	77.0%	82.2%	93.4%
**Relative Force at Minimal Displacement [% BW]**	348	69.8%	69.2%	70.4%	5.6%	50.6%	59.3%	62.8%	66.2%	69.8%	73.1%	76.6%	81.4%	93.4%
**Braking Rate of Force Development [N/s]**	348	42.5%	41.2%	43.7%	12.1%	17.1%	23.1%	28.2%	34.5%	41.6%	49.2%	58.7%	69.4%	87.6%
**Average Braking Force [N]**	348	76.5%	75.9%	77.1%	5.7%	58.9%	65.8%	69.6%	72.8%	76.2%	80.1%	83.4%	87.1%	100.2%
**Average Relative Braking Force [% BW]**	348	76.3%	75.7%	76.9%	5.6%	58.8%	65.8%	69.6%	72.7%	76.0%	80.0%	83.1%	86.6%	95.9%
**Peak Braking Force [N]**	348	70.1%	69.5%	70.7%	5.7%	50.5%	59.8%	63.2%	66.4%	70.2%	73.2%	77.4%	81.7%	90.1%
**Peak Relative Braking Force [% BW]**	348	70.0%	69.4%	70.5%	5.6%	50.6%	59.6%	63.1%	66.4%	70.1%	73.1%	77.0%	81.3%	88.8%
**Average Propulsive Force [N]**	348	75.6%	75.2%	76.1%	4.2%	61.6%	68.4%	71.0%	73.1%	75.5%	78.0%	80.9%	83.3%	101.3%
**Average Relative Propulsive Force [% BW]**	348	75.5%	75.1%	75.9%	3.9%	61.5%	68.4%	70.8%	72.9%	75.4%	77.8%	80.7%	83.3%	89.0%
**Peak Propulsive Force [N]**	348	72.3%	71.7%	72.9%	5.8%	50.8%	62.3%	65.7%	68.5%	72.4%	75.7%	79.4%	83.5%	101.4%
**Peak Relative Propulsive Force [% BW]**	348	72.2%	71.6%	72.8%	5.7%	50.8%	62.2%	65.5%	68.5%	72.2%	75.6%	79.2%	83.0%	101.2%
**Average Braking Power [W]**	348	52.9%	52.0%	53.8%	8.2%	28.3%	37.1%	41.8%	48.0%	53.2%	58.3%	61.9%	67.9%	81.8%
**Average Relative Braking Power [W/kg]**	348	52.8%	51.9%	53.7%	8.1%	27.9%	37.1%	41.8%	47.9%	53.1%	58.2%	61.7%	67.5%	81.8%
**Peak Braking Power [W]**	348	53.5%	52.6%	54.4%	8.9%	26.7%	36.7%	41.8%	47.3%	53.5%	59.5%	64.6%	69.7%	79.4%
**Peak Relative Braking Power [W/kg]**	348	53.4%	52.5%	54.3%	8.8%	26.4%	36.6%	41.8%	47.3%	53.4%	59.4%	64.4%	69.5%	79.2%
**Average Propulsive Power [W]**	348	53.6%	53.1%	54.0%	4.3%	39.5%	46.3%	48.6%	50.9%	53.3%	56.1%	58.7%	61.5%	79.4%
**Average Relative Propulsive Power [W/kg]**	348	53.4%	53.0%	53.9%	4.1%	39.5%	46.2%	48.6%	50.9%	53.2%	56.1%	58.6%	61.4%	67.7%
**Peak Propulsive Power [W]**	348	56.4%	55.9%	56.9%	4.7%	45.8%	48.6%	50.4%	53.5%	56.0%	58.8%	61.9%	65.3%	83.8%
**Peak Relative Propulsive Power [W/kg]**	348	56.3%	55.8%	56.8%	4.5%	45.7%	48.6%	50.3%	53.4%	56.0%	58.8%	61.7%	65.1%	73.6%
**Average Braking Velocity [m/s]**	348	66.2%	65.3%	67.0%	8.0%	40.3%	48.7%	55.3%	61.5%	66.9%	71.6%	74.9%	78.9%	88.3%
**Peak Braking Velocity [m/s]**	348	67.6%	66.7%	68.5%	8.6%	41.1%	49.0%	55.6%	63.0%	68.8%	72.9%	77.5%	82.1%	90.7%
**Average Propulsive Velocity [m/s]**	348	69.5%	69.1%	69.9%	3.9%	49.2%	62.4%	64.4%	67.0%	69.9%	72.3%	74.0%	75.7%	80.6%
**Peak Velocity [m/s]**	348	70.1%	69.8%	70.5%	3.4%	57.7%	63.6%	65.8%	67.7%	70.3%	72.4%	74.1%	76.0%	79.0%
**Takeoff Velocity [m/s]**	348	66.4%	65.9%	66.8%	4.4%	47.4%	58.4%	60.6%	63.5%	66.9%	69.6%	71.3%	73.3%	76.2%
**Positive Impulse [N**⋅**s]**	348	95.6%	94.9%	96.3%	6.8%	73.3%	83.6%	87.7%	91.3%	95.6%	99.7%	103.2%	108.3%	122.2%
**Positive Net Impulse [N**⋅**s]**	348	67.0%	66.5%	67.5%	4.9%	50.0%	56.5%	60.3%	64.0%	67.5%	70.4%	72.6%	74.6%	89.4%
**Braking Impulse [N**⋅**s]**	348	96.9%	96.0%	97.9%	9.3%	65.1%	79.7%	85.3%	91.3%	97.4%	102.6%	107.3%	114.6%	128.1%
**Braking Net Impulse [N**⋅**s]**	348	67.8%	66.9%	68.8%	8.7%	41.6%	49.1%	55.7%	63.0%	69.0%	73.2%	77.6%	82.7%	91.4%
**Relative Braking Impulse [N**⋅**s/kg]**	348	96.8%	95.8%	97.7%	9.3%	65.4%	79.1%	85.4%	91.0%	97.0%	102.3%	107.2%	114.3%	127.8%
**Relative Braking Net Impulse [N**⋅**s/kg]**	348	67.7%	66.8%	68.6%	8.6%	41.0%	49.1%	55.6%	63.0%	68.9%	73.0%	77.6%	82.3%	90.9%
**Propulsive Impulse [N**⋅**s]**	348	94.8%	94.2%	95.5%	6.3%	74.4%	83.8%	87.3%	90.8%	94.7%	98.6%	101.6%	107.3%	125.4%
**Propulsive Net Impulse [N**⋅**s]**	348	66.6%	66.1%	67.0%	4.5%	47.7%	58.7%	60.9%	63.6%	67.1%	69.6%	71.6%	73.5%	91.5%
**Relative Propulsive Impulse [N**⋅**s/kg]**	348	94.7%	94.0%	95.3%	6.1%	74.3%	83.6%	87.2%	90.8%	94.6%	98.4%	101.3%	106.2%	125.2%
**Relative Propulsive Net Impulse [N**⋅**s/kg]**	348	66.4%	66.0%	66.9%	4.4%	47.7%	58.7%	60.7%	63.5%	67.0%	69.6%	71.4%	73.2%	76.5%
**Impulse Ratio [a.u.]**	348	100.2%	98.7%	101.7%	14.2%	73.4%	80.7%	85.8%	91.3%	97.8%	106.8%	116.9%	130.4%	196.4%

Legend: N—sample size; SD—standard deviation; CI—confidence intervals; P—percentile; mRSI—modified reactive strength index; BW—body weight; a.u.—arbitrary units. Note: All values represent athlete-level unilateral-to-bilateral ratios calculated as the mean unilateral value, averaged across the left and right limbs, divided by the mean bilateral value and multiplied by 100.

## Data Availability

The full script and data are available as Supplementary Material in a public repository via Zenodo https://doi.org/10.5281/zenodo.18710843.

## Supplementary Materials

The following supporting information can be downloaded at: https://www.mdpi.com/article/10.3390/sports14080323/s1. Table S1: Force Plate Protocol—Warm-Up; Table S2. Operational Definitions of Kinetic, Kinematic, and Temporal Metrics Extracted from the Vertical Ground Reaction Force Signal; Table S3. Sex-Based Differences; Table S4. Male Reference Data; Table S5. Female Reference Data; Table S6. Shapiro–Wilk Normality Tests.

## Abbreviations

ACL	anterior cruciate ligament
a.u.	arbitrary units
BD	bilateral deficit
BF	bilateral facilitation
BW	body weight
CI	confidence interval
CMJ	bilateral countermovement jump
CV%	coefficient of variation expressed as a percentage
mRSI	modified reactive strength index
N	sample size
P	percentile
pp	percentage points
RFD	rate of force development
RTS	return-to-sport
SD	standard deviation
SL CMJ	single-leg countermovement jump
SSC	stretch-shortening cycle
